# Convenient methods for ingestion of molecular hydrogen: drinking, injection, and inhalation

**DOI:** 10.1186/s13618-015-0034-2

**Published:** 2015-10-26

**Authors:** Ryosuke Kurokawa, Tomoki Seo, Bunpei Sato, Shin-ichi Hirano, Fumitake Sato

**Affiliations:** MiZ Co., Ltd., 2-19-15 Ofuna, Kamakura, Kanagawa 247-0056 Japan

**Keywords:** Hydrogen water, Hydrogen-rich saline, Hydrogen gas

## Abstract

Molecular hydrogen (H_2_) is clinically administered; however, in some hospitals, H_2_ is given to patients without consideration of its safe use. In the present study, we prepared convenient and safe devices for the drinking of super-saturated H_2_ water, for intravenous drip infusion of H_2_-rich saline, and for the inhalation of H_2_ gas. In order to provide useful information for researchers using these devices, the changes in H_2_ concentration were studied. Our experimental results should contribute to the advance of non-clinical and clinical research in H_2_ medicine.

## Background

Molecular hydrogen (H_2_) is a medical gas with beneficial effects on oxidative stress [1], inflammation [2], apoptosis [3], lipid metabolism [4], and signaling pathways [5]. More than 280 articles, including 24 articles on clinical studies, have demonstrated that H_2_ ameliorates the pathological conditions in numerous human diseases [6] or disease models in animals [7], since Ohsawa et al. reported that H_2_ could be used in antioxidant therapy [8].

H_2_ is clinically administered through the oral intake of H_2_ water [9–12], intravenous drip infusion of H_2_-rich saline [12–15], or inhalation of air with 2-4 % H_2_ gas [12]. However, in some hospitals, H_2_ is given to patients by intravenous drip infusion and/or inhalation without consideration of its safe use. We have developed and provided various devices for the ingestion of H_2_ to solve this problem. Furthermore, the beneficial effects of H_2_ using our devices have been reported in 7 human diseases [9–16].

In the present study, we prepared convenient and safe devices for drinking super-saturated H_2_ water, for intravenous drip infusion of H_2_-rich saline, and for the inhalation of H_2_ gas. We examined the changes in H_2_ concentrations in these devices in order to provide useful information for researchers. Our experimental results reported in this article should contribute to the advance of non-clinical and clinical research in H_2_ medicine.

## Methods/design

### Materials

A pressure-resistant 500 mL PET bottle (e.g., a Coke bottle) was used. H_2_-generating agent (0.65 g) was prepared by mixing aluminum powder and calcium hydroxide at a ratio of 76 to 24 by weight. The agent was entirely wrapped with bags, namely, a gas-permeable film or non-woven fabric. The wrapped agent was then reacted with water to generate H_2_ as follows:

$$ 2\mathrm{A}\mathrm{l} + \mathrm{C}\mathrm{a}\ {\left(\mathrm{O}\mathrm{H}\right)}_2 + 6{\mathrm{H}}_2\mathrm{O}\ \to\ \mathrm{C}\mathrm{a}\ {\left[\mathrm{A}\mathrm{l}\ {\left(\mathrm{O}\mathrm{H}\right)}_4\right]}_2 + 3{\mathrm{H}}_2. $$

### Preparation of super-saturated H_2_ water for drinking

#### Method I

**Fig. 1:**
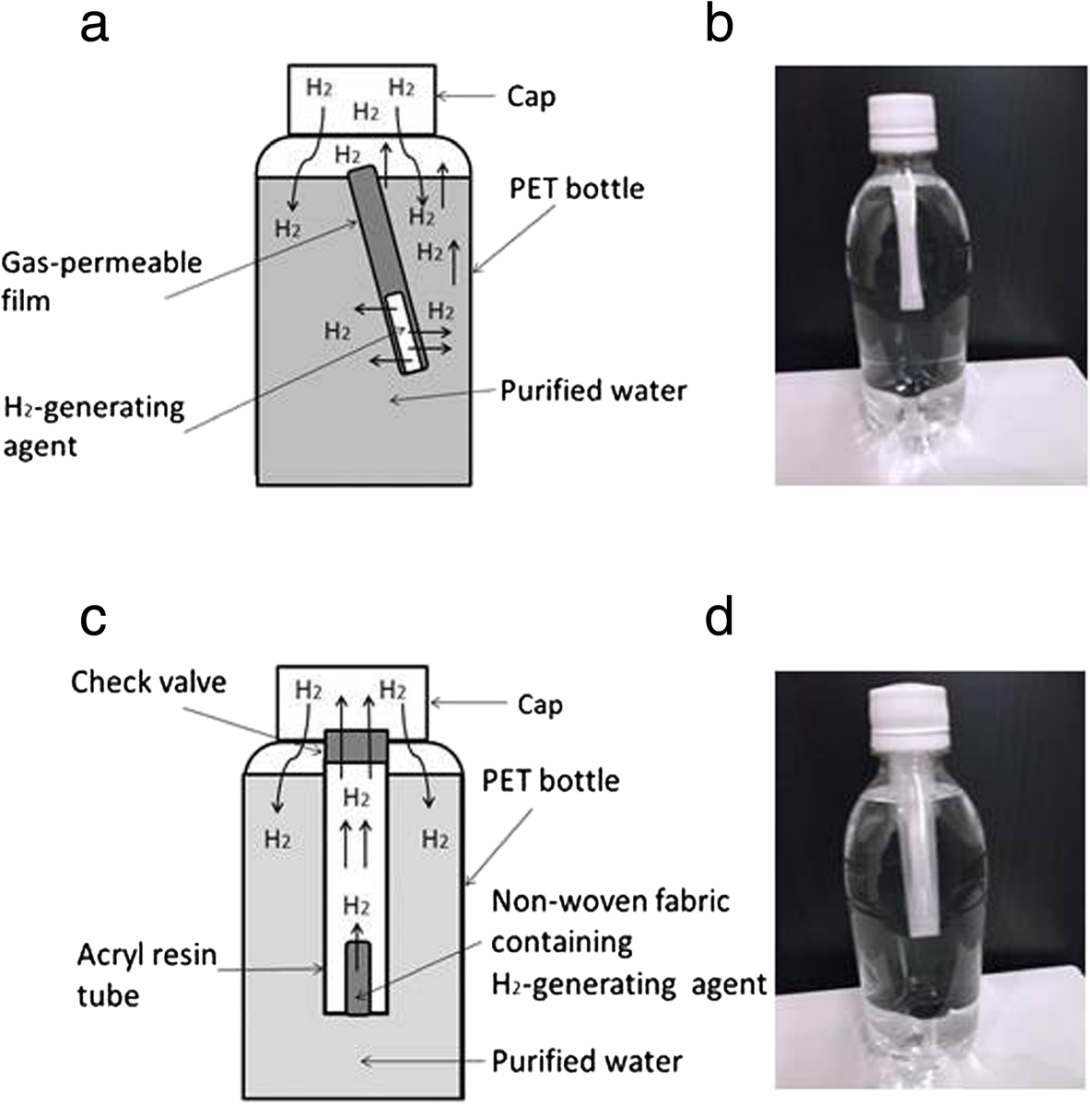
Devices for super-saturated H_2_ water. **a** and **b** Method I: A PET bottle, in which a gas-permeable film containing H_2_-generating agent has been directly inserted, is filled with water and then tightly closed. The H_2_ gas produced is emitted into the water in the bottle, lowering the height of the water level, which is then gradually pressurized by the gas. After the reaction is terminated, the H_2_ gas is dissolved by shaking the bottle. **c** and **d** Method II: The non-woven fabric containing H_2_-generating agent is first inserted into an acrylic resin tube, and 0.5 mL of water is added. The tube is inserted into a PET bottle filled with water. The H_2_ gas generated in the tube is then transferred to the bottle through the valve

As shown in Fig. 1a and b, a pressure-resistant PET bottle (500 mL), in which gas-permeable film had been directly inserted, was filled with water and then tightly closed. Water in the bottle reacted with the H_2_-generating agent (0.65 g), and the H_2_ gas produced was emitted into the water in the bottle through the gas-permeable film. Thus, during this procedure, the H_2_-generating agent as well as the water for the reaction did not come into contact with the drinking water. During the reaction, the H_2_ gas reduced the height of the water level in the standing bottle, which was gradually pressurized to approximately 4.5 atmospheric pressures by the gas after 24 h at room temperature. After the reaction was terminated, the H_2_ gas was dissolved by shaking the bottle for about 30 s.

#### Method II

Similarly, H_2_ water was obtained by the use of non-woven fabric. As shown in Fig. 1c and d, the non-woven fabric containing H_2_-generating agent (0.65 g) was first inserted into an acrylic resin tube, and 0.5 mL of water was added. The tube was tightly closed with a cap attached to a check valve, and inserted into a pressure-resistant PET bottle filled with water. H_2_ generated in the tube was transferred to the bottle through the valve. In about 5 min at room temperature, the agent started a reaction in the wet fabric. The H_2_ gas produced was emitted into the water through the check valve attached to the acrylic resin tube. During the reaction, the PET bottle was gradually pressurized to approximately 6 atmospheric pressures due to the generation of H_2_ gas. After 24 h, the H_2_ gas was dissolved by shaking the bottle for about 30 s.

### Preparation of H_2_-rich saline for injection

#### Method III

**Fig. 2:**
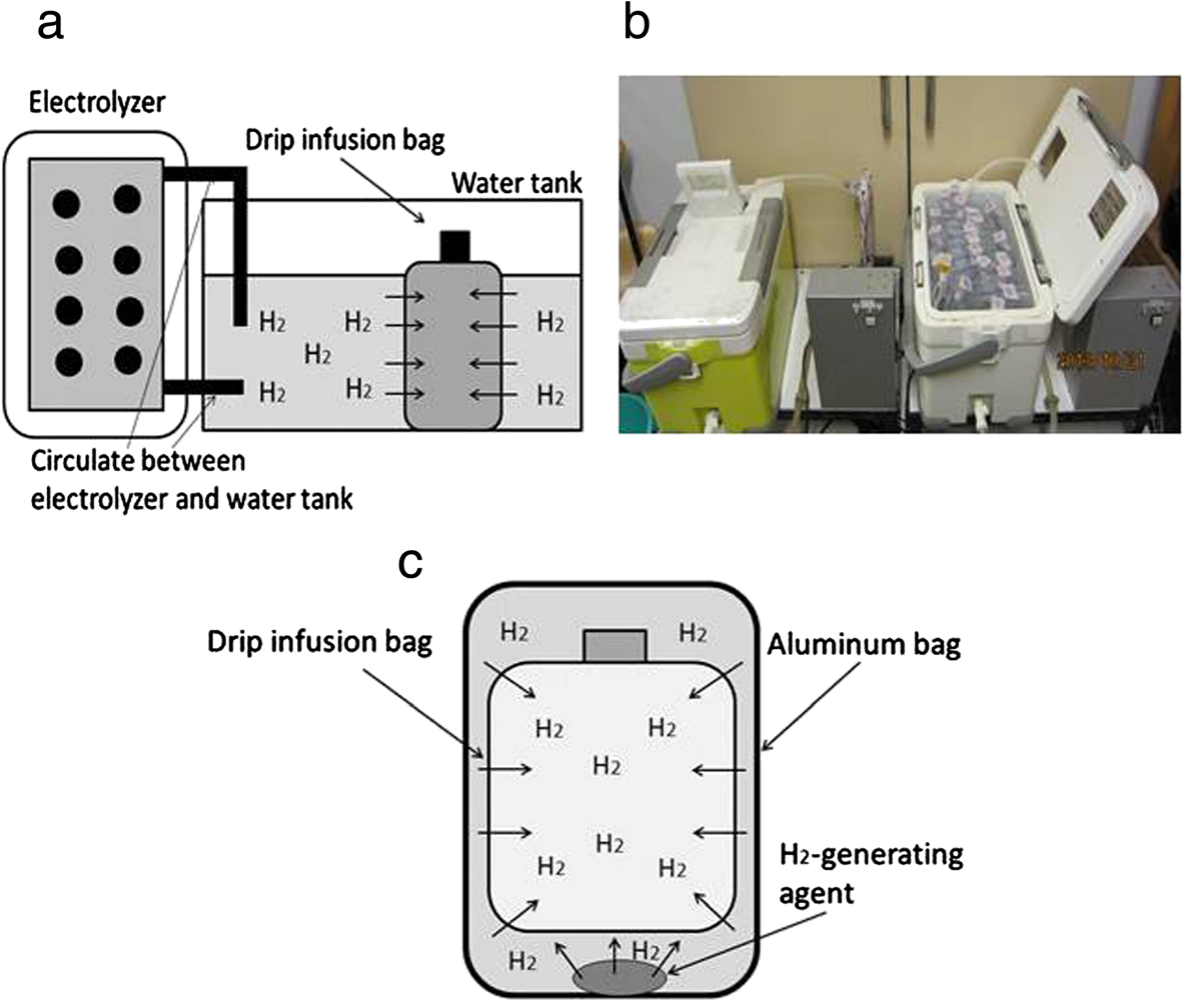
Devices for H_2_-rich saline. **a** and **b** Method III: A polyethylene bag for drip infusion is immersed in an H_2_-containing water tank where the water is continuously electrolyzed and circulated during operation. H_2_ permeates through the polyethylene film and is dissolved into the solution without contamination. **c** Method IV: Non-woven fabric containing the H_2_-generating agent is moistened with a small amount of water, and then both a drip infusion bag and non-woven fabric are wrapped with aluminum foil under reduced pressure. The water reacts with the agent in the non-woven fabric to generate H_2_, and the H_2_ gas permeating through the polyethylene film in the bag dissolves into the solution

As shown in Fig. 2a and b, a polyethylene bag for drip infusion, dialysis fluid, or organ storage solution was immersed in a H_2_-containing water tank where the water was continuously electrolyzed and circulated during the operation. The H_2_ permeated through the polyethylene film and dissolved in the solution without contamination.

#### Method IV

As shown in Fig. 2c, non-woven fabric containing the H_2_-generating agent was moistened with a small amount of water, and then both a drip infusion bag and the non-woven fabric were wrapped with aluminum foil under reduced pressure. The water reacted with the agent in the non-woven fabric to generate H_2_, and the H_2_ gas permeating through the polyethylene film in the bag dissolved into the solution.

### Preparation of H_2_-containing gas for inhalation

**Fig. 3:**
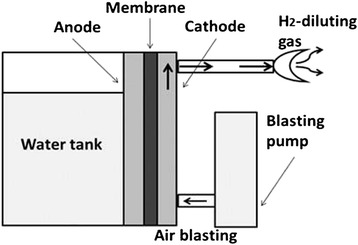
Apparatus for H_2_ gas inhalation. The inhalation gas is prepared by mixing H_2_ gas and air, in which the H_2_ gas was produced by the electrolysis of water, and the concentration is controlled under the detonation limit of the mixture of H_2_ gas and air

As shown in Fig. 3, inhalation gas was prepared by the mixing of H_2_ gas and air, where the H_2_ gas was produced by the electrolysis of water, and the concentration was controlled under the detonation limit of the mixture of H_2_ gas and air (below 4 %).

### Measurement of H_2_ concentration

The concentration of H_2_ gas in the water was measured using the methylene blue platinum colloid reagent-based titration method, as described previously [17], and verified using an electrochemical gas sensor (model DHD1-1, DKK-TOA Corp., Tokyo, Japan). On the other hand, the concentration of H_2_ in the air was measured using an H_2_ gas sensor (FIS Inc., Hyogo, Japan).

### Statistical analysis

The concentration of H_2_ gas in the water or air is presented as ppm (mg/L, weight/volume) or % (volume/volume), respectively. Most of the experimental data are expressed as mean ± standard deviation (mean ± SD) of more than three individual measurements. However, in the examination of H_2_-rich saline, the H_2_ concentration is expressed as an individual measurement to examine the differences between each bag and plastic vessel. The statistical significance was assessed by Student’s paired or unpaired *t*-test for single comparisons or by one-way analysis of variance (ANOVA) followed by Fisher’s LSD test for multiple comparisons. A *p* value of less than 0.05 was considered to be statistically significant.

## Results/discussion

### H_2_ concentration of super-saturated H_2_ water prepared by Method I

**Fig. 4:**
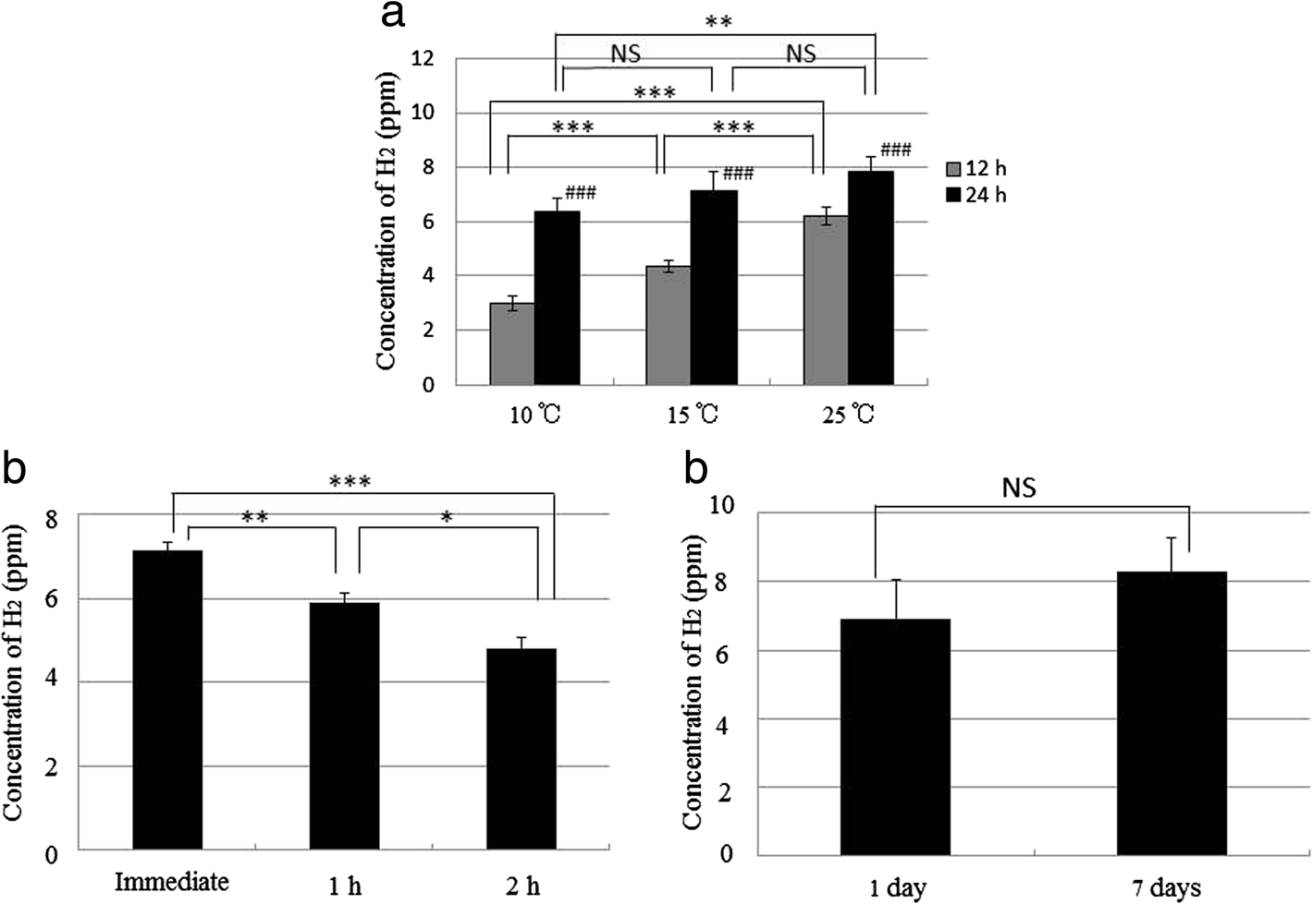
Concentrations of H_2_ in the super-saturated H_2_-rich water prepared by Method I. **a** Concentrations of H_2_ measured at 10, 15, and 25 °C after 12 and 24 h (###*p* < 0.001, 12 h vs. 24 h at the same temperature; ***p < 0.001, 10 °C vs. 15 °C after 12 h, 15 °C vs. 25 °C after 12 h, or 10 °C vs. 25 °C after 12 h; **p < 0.01, 10 °C vs. 25 °C after 24 h). **b** Concentrations of H_2_ measured immediately after 24 h, and then measured 1 or 2 h after the cap had been opened (***p < 0.001, Immediate vs. 2 h; **p < 0.01, Immediate vs. 1 h; *p < 0.05, 1 h vs. 2 h). **c** Concentrations of H_2_ measured after 1 or 7 days without opening. Data are presented as mean ± standard deviation (SD) for 3–5 independent measurements

H_2_ concentrations in the super-saturated H_2_ water prepared by Method I were measured at 10 °C, 15 °C, and 25 °C. As shown in Fig. 4a, at the same temperature, each H_2_ concentration after 24 h was significantly increased compared with each H_2_ concentration after 12 h (*p* < 0.001). After 12 h, H_2_ concentration at 25 °C was significantly increased compared with the concentration at 15 °C (*p* < 0.001), and the concentration at 15 °C was significantly increased compared with that at 10 °C (*p* < 0.001). In addition, after 24 h, H_2_ concentration at 25 °C showed a significant increase compared with that at 10 °C (*p* < 0.01). The H_2_ concentration after the opening of the PET bottle was also measured at room temperature. As shown in Fig. 4b, H_2_ concentration of the water was maintained at approximately 7 ppm (7.13 ± 0.22 ppm) after 24 h without opening the bottle; after the cap had been opened, the concentration after 1 h was significantly decreased compared with the concentration after immediately opening (*p* < 0.01). In addition, H_2_ concentration after 2 h was significantly decreased compared with that after 1 h (*p* < 0.05). In our preliminary experiment after opening the bottle, the H_2_ concentrations in the bottle after 1 and 3 h were 4.53 ± 0.15 ppm and 2.10 ± 0.10 ppm (each *n* = 3), respectively, when 150 mL of water was removed immediately after the termination of H_2_ gas production, and the same volume of water additionally removed after 1 h (data not shown). Furthermore, to examine the stability without opening, H_2_ concentration was measured after 7 days. As shown in Fig. 4c, the H_2_ concentration of the water was maintained above 8 ppm (8.30 ± 0.98 ppm) after 7 days without the opening of the bottle. These results suggest that the H_2_ concentration is maintained for at least 7 days without opening, but the H_2_ water should be drunk within 2 h after opening. In addition, it is important that after opening, the bottle should not contain space for air in order to avoid the reduction of H_2_ concentration.

### H_2_ concentration of super-saturated H_2_ water prepared by Method II

**Fig. 5:**
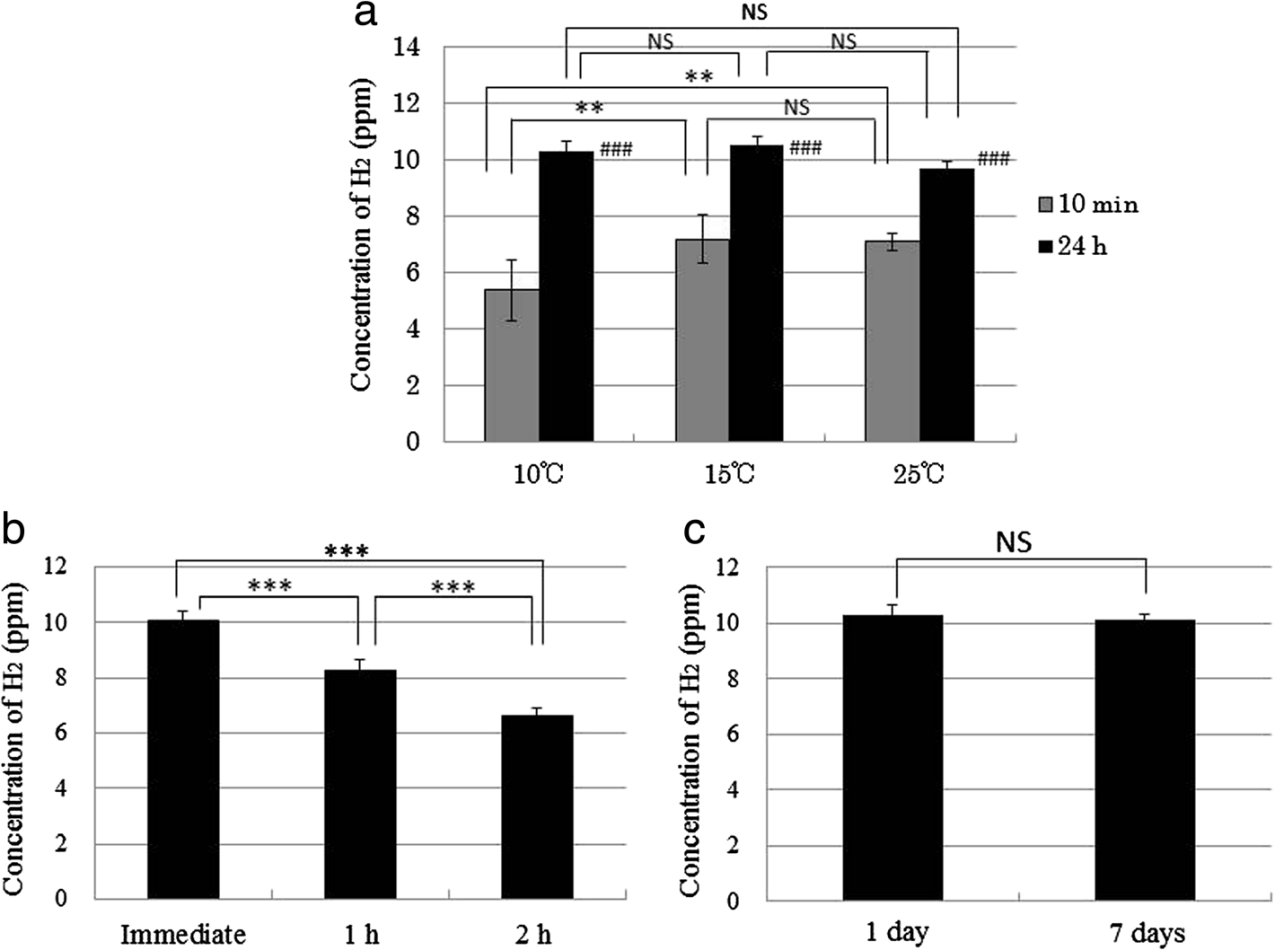
Concentrations of H_2_ in the super-saturated H_2_-rich water prepared by Method II. **a** Concentrations of H_2_ measured at 10, 15, and 25 ^∘^C after 10 min and 24 h (###*p* < 0.001, 10 min vs. 24 h at the same temperature; **p < 0.01, 10 ^∘^C vs. 15 ^∘^C after 10 min, or 10 ^∘^C vs. 25 ^∘^C after 10 min). **b** Concentrations of H_2_ measured immediately after 24 h, and then measured 1 or 2 h after the cap had been opened (***p < 0.001, Immediate vs. 1 h, 1 h vs. 2 h, or Immediate vs. 2 h). **c** Concentrations of H_2_ measured after 1 or 7 days without opening. Data are presented as mean ± standard deviation (SD) for 3–5 independent measurements

H_2_ concentrations in the super-saturated H_2_ water prepared by Method II were also measured at 10 °C, 15 °C, and 25 °C. As shown in Fig. 5a, at the same temperature, each H_2_ concentration after 24 h was significantly increased compared with each H_2_ concentration after 10 min (*p* < 0.001). After 10 min, H_2_ concentration at 15 °C was significantly increased compared with the concentration at 10 °C (*p* < 0.01), and the concentration at 25 °C was significantly increased compared with that at 10 °C (*p* < 0.01). As shown in Fig. 5b, H_2_ concentration of the water was maintained at approximately 10 ppm (10.08 ± 0.34 ppm) after 24 h without opening of the bottle; after the cap had been opened, the concentration after 1 h showed significant decrease compared with that after immediately opening (*p* < 0.001), and the concentration after 2 h also showed significant decrease compared with that after 1 h (*p* < 0.001). As shown in Fig. 5c, the H_2_ concentration of the water was maintained at approximately 10 ppm (10.10 ± 0.21 ppm) after 7 days without opening of the bottle. These results suggest that the H_2_ concentration prepared by this method is maintained for at least 7 days without opening, but the water should be drunk within 2 h of the cap being opened.

### H_2_ concentration of H_2_-rich saline prepared by Method III

**Fig. 6:**
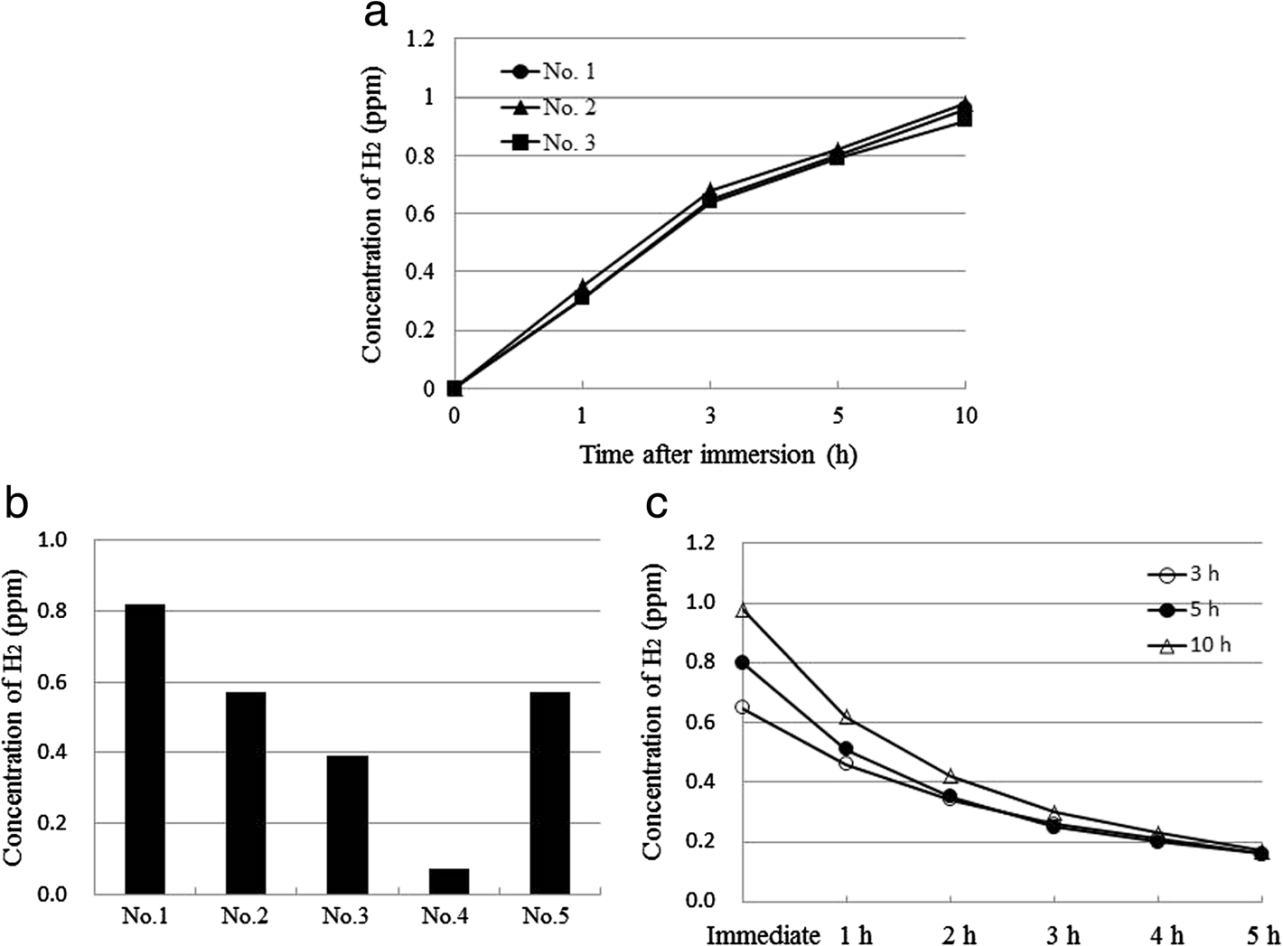
Concentrations of H_2_ in the H_2_-rich saline prepared by Method III. **a** Concentrations of H_2_ measured at 1, 3, 5, and 10 h after immersion of the drip infusion bags. **b** Concentrations of H_2_ measured at 5 h after the immersion of each infusion bag and polyethylene vessel. **c** Concentrations of H_2_ measured at 1, 2, 3, 4, and 5 h after removal from the bath. Data are presented as individual measurements

The H_2_ concentrations of H_2_-rich saline prepared by Method III in the infusion bags were measured after immersion for 1, 3, 5, and 10 h (Table 1). When the 3 types of bag (No. 1–3) were immersed for 10 h, approximately 1.0 ppm H_2_-rich saline was obtained (Fig. 6a). There were no differences in the H_2_ concentration between the types of drip infusion bag. These results demonstrated that it is necessary to immerse the drip infusion bag for at least 10 h in order to obtain 1.0 ppm H_2_-rich saline. To examine the permeability of H_2_ for the different polyethylene vessel materials, 5 types of vessels (No. 1–5) were immersed in the water bath for 5 h, and the change in H_2_ concentration of each vessel was examined (Table 1). The H_2_ concentration of various infusion bags and polyethylene vessels depends on their thickness and the content of the solution. The H_2_ easily penetrated into the physiological saline (No. 1), but barely penetrated into the sodium hydrogen carbonate solution (No. 4). In addition, in the physiological saline, the H_2_ more easily penetrated into the 500 mL drip infusion bag (No. 1) than the 20 mL plastic injection ampoule (No. 5) (Fig. 6b). After the infusion bags had been immersed in the bath for 3, 5, and 10 h, they were removed and the changes in H_2_ concentration were measured until 5 h later. The H_2_ concentration of the drip infusion bag decreased from 1.0 ppm to 0.6 ppm after 1 h of removal from the water bath after immersion for 10 h (Fig. 6c). These results suggest that intravenous drip injection with these bags should be completed within 1 h.

**Table 1:** Details of drip infusion bag, dialysis fluid bag, and injection ampoule used in the experiment

Experiment	No.	Trade name	Volume (mL)	Purpose	Vendor
A	1	5 % Glucose injection	500	DI	T
	2	Solulact (Lactate ringer sol.)	500	DI	T
	3	Isotonic sodium chloride sol.	500	DI	T
B	1	Otsuka normal saline	500	DI	O
	2	Hartman’s sol. pH 8 (Lactate ringer sol.)	500	DI	N
	3	5 % Glucose injection (for animals)	500	DI	K
	4	7 % Sodium hydrogen carbonate sol. (for animals)	500	DI	K
	5	Otsuka normal saline	20	I	O
C	1	Otsuka normal saline	500	DI	O
	2	Midperiq	2,000	DF	T
	3	Isotonic sodium chloride sol.	100	DI	T
	4	Isotonic sodium chloride sol.	500	DI	T

A: Time-dependent concentration after immersion, B: Difference between types of containers, C: Storage stability in aluminum bag, sol.: Solution, DI: Drip infusion, I: Injection, DF: Dialysis fluid, T: Terumo Corp., Tokyo, Japan, O: Otsuka Pharmaceutical Co., Ltd., Tokyo, Japan, N: Nipro Corp., Osaka, Japan, K: Kyoritsu Seiyaku Corp., Tokyo, Japan

### H_2_ concentration of H_2_-rich saline prepared by Method IV

**Fig. 7:**
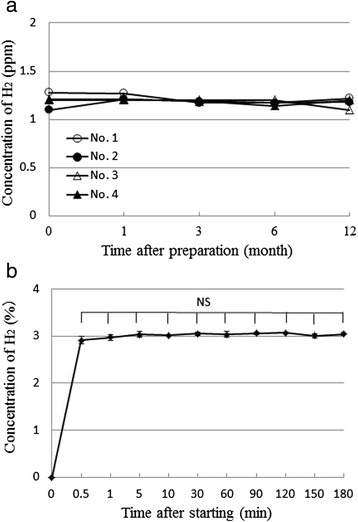
Concentrations of H_2_ in the devices. **a** Concentrations of H_2_ in the H_2_-rich saline were measured at 1, 3, 6 and 12 months after preparation by Method IV. **b** Concentrations of H_2_ in the H_2_ gas inhaler were measured up to 3 h after starting. Data are presented as (**a**) individual measurements or (**b**) mean ± standard deviation (SD) for 3 independent measurements

The H_2_ concentrations of 4 types of bag (No. 1–4) prepared by Method IV were also measured after 1, 3, 6, and 12 months in order to examine long-term preservation (Table 1). The H_2_ concentrations in the drip infusion bags (No. 1, 3, and 4) or dialysis fluid bag (No. 2) were maintained for 12 months, suggesting that the H_2_-rich saline prepared by this method could be used for 12 months (Fig. 7a).

### H_2_ concentration of gas introduced by inhaler

We examined the H_2_ gas concentration for up to 3 h after starting use of the inhaler, because stability of the gas concentration is required in order to examine the performance of the gas inhaler. The H_2_ gas concentration in the inhaler was 2.91 ± 0.08 % after 0.5 min, and a H_2_ gas concentration of approximately 3 % was maintained for 3 h. There was no significant difference among of the time points after starting (Fig. 7b). These results demonstrate that the H_2_ gas could be supplied stably for 3 h using this inhaler.

In summary, we prepared two types of super-saturated H_2_ water (7 or 10 ppm) for drinking. The concentrations in these waters were maintained for 7 days without opening, but the waters should be drunk within 2 h of the cap being opened. We also prepared two types of H_2_-rich saline for injection. Although intravenous drip injection with the H_2_-rich saline should be completed within 1 h, H_2_ concentrations in the saline prepared by aluminum foil (Method IV) were maintained for 12 months without opening. Moreover, we prepared H_2_-containing gas for inhalation. The gas was controlled under the detonation limit of the mixture of H_2_ gas and air, and the gas could be supplied stably for 3 h. In a recent study, we examined the H_2_ concentration in rat tissue following administration of H_2_ via various routes [18]. We demonstrated that H_2_ concentrations in the tissues depend on the H_2_ concentration of the administered water or gas, and that the specific uptake of H_2_ in the tissues is due to the difference in administration route [18]. The present results suggest the importance in choosing the more efficient route of H_2_ treatment for each disease or tissue [18]. Therefore, we believe that the super-saturated H_2_ water (10 ppm) prepared by Method II, the H_2_-rich saline prepared by Method IV, and the H_2_ gas prepared by our method are convenient and safe preparatory methods. The present results should contribute to the advance of non-clinical and clinical research in H_2_ medicine.

## Abbreviations

H_2_: molecular hydrogen
sol.: solution
